# IceLines – A new data set of Antarctic ice shelf front positions

**DOI:** 10.1038/s41597-023-02045-x

**Published:** 2023-03-15

**Authors:** Celia A. Baumhoer, Andreas J. Dietz, Konrad Heidler, Claudia Kuenzer

**Affiliations:** 1 Earth Observation Center (EOC), German Aerospace Center (DLR), Weßling, Germany; 2 Data Science in Earth Observation, Technical University of Munich (TUM), Munich, Germany; 3 Institute of Geography and Geology, University Wurzburg, Wurzburg, Germany

**Keywords:** Environmental impact, Cryospheric science, Geomorphology

## Abstract

The frontal position of an ice shelf is an important parameter for ice dynamic modelling, the computation of mass fluxes, mapping glacier area change, calculating iceberg production rates and the estimation of ice discharge to the ocean. Until now, continuous and up-to-date information on Antarctic calving front locations is scarce due to the time-consuming manual delineation of fronts and the previously limited amount of suitable earth observation data. Here, we present IceLines, a novel data set on Antarctic ice shelf front positions to assess calving front change at an unprecedented temporal and spatial resolution. More than 19,400 calving front positions were automatically extracted creating dense inter- and intra-annual time series of calving front change for the era of Sentinel-1 (2014-today). The calving front time series can be accessed via the EOC GeoService hosted by DLR and is updated on a monthly basis. For the first time, the presented IceLines data set provides the possibility to easily include calving front dynamics in scientific studies and modelling to improve our understanding about ice sheet dynamics.

## Background & Summary

Three-quarters of the Antarctic coastline consist of floating ice shelves regulating the ice discharge of the Antarctic Ice Sheet (AIS)^1^. Retreating or even disintegrating ice shelves can reduce buttressing effects resulting in enhanced mass loss of the AIS^2^. Within the last decades, disintegration events along the Antarctic Peninsula (AP) and the West Antarctic Ice Sheet (WAIS) demonstrated the dynamic behavior and vulnerable state of Antarctic ice shelves^3–5^. In total since 1997, Antarctic ice shelf area loss due to disintegration and calving events has been dominant compared to ice shelf area gain by advancing fronts^6,7^. The growth and decay of an ice shelf is controlled by several factors such as internal ice dynamics, ice shelf geometry, pinning points, bed topography and external mechanical and climatic forces. There is growing evidence that long-term atmospheric and ocean forcing cause ice shelf front retreat along the AP and WAIS^3,4,8–10^. For the East Antarctic Ice Sheet (EAIS), the body of evidence is not as clear as calving front time series are shorter and less frequent^7,11–13^. The calving front position is of significant value to Antarctic studies focusing on oceanography, sea ice, glaciology and terrestrial or ocean ecology. Especially in glaciology, the front position is an important parameter for ice dynamic modelling^14^, computing mass fluxes^15^, mapping glacier area change^8^, calculating the iceberg production rate^16^ and estimating the export of ice mass to the ocean^17^. Therefore, knowledge about sub-annual calving front dynamics and their short-term controlling mechanisms are essential for a better understanding of ice sheet dynamics determining the mass loss or gain of the AIS^12,15^. But so far, such data does not exist for the entire AIS due to time-consuming manual front delineations and the previously limited availability of satellite imagery^12^. To overcome the tedious manual work of calving front delineation, traditional imaging techniques have been used to develop automated approaches not suitable for intra-annual calving front extraction^18–20^ due to seasonal variations in sea ice and surface melt. To date, only deep learning based approaches are able to provide accurate and dense time series of calving front location change^21–23^.

**Fig. 1:**
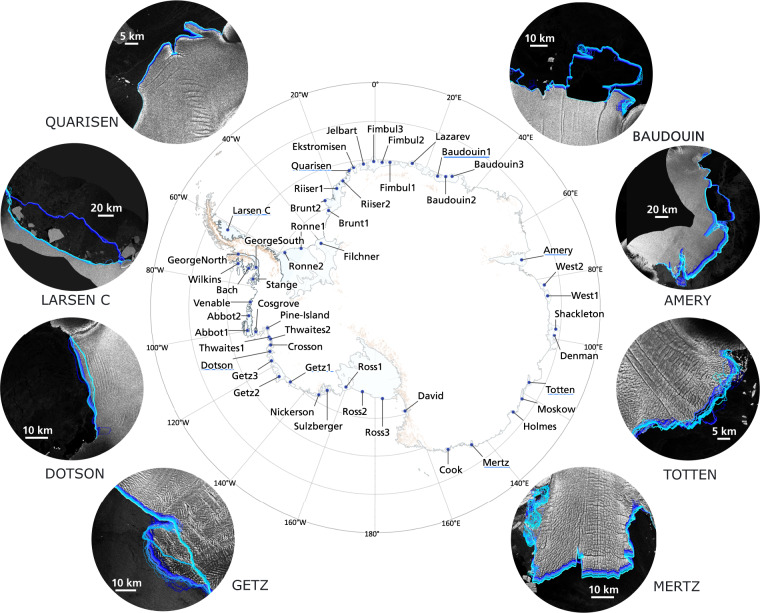
Overview of all ice shelf fronts continuously monitored with IceLines. Exemplary ice shelf front time series generated with IceLines are shown at the sides. Copernicus Sentinel-1 Data 2022.

We take advantage of these innovative techniques and introduce IceLines^24^, a data set of Antarctic ice shelf front positions providing continuous and up-to-date calving front time series. IceLines is a novel deep learning-based framework providing calving front locations (CFL) on different temporal scales (daily, monthly, quarterly, annual) for Antarctic ice shelves automatically extracted from Sentinel-1 radar imagery. The data set includes all intact Antarctic ice shelves listed by the SCAR Composite Gazetter^25^ with a width of at least 30 km. Additionally, six dynamic glacier fronts being of key interest (e.g. Pine Island Glacier) have been selected for monitoring. Figure 1 shows the ice shelf fronts and glacier tongues currently monitored by IceLines. Depending on the Sentinel-1 data availability, the provided calving front time series covers the time period from 2014 until today and is automatically updated on a monthly basis. The IceLines data^24^ is freely available via DLR’s GeoService (https://geoservice.dlr.de/web/maps/eoc:icelines).

## Methods

**Fig. 2:**
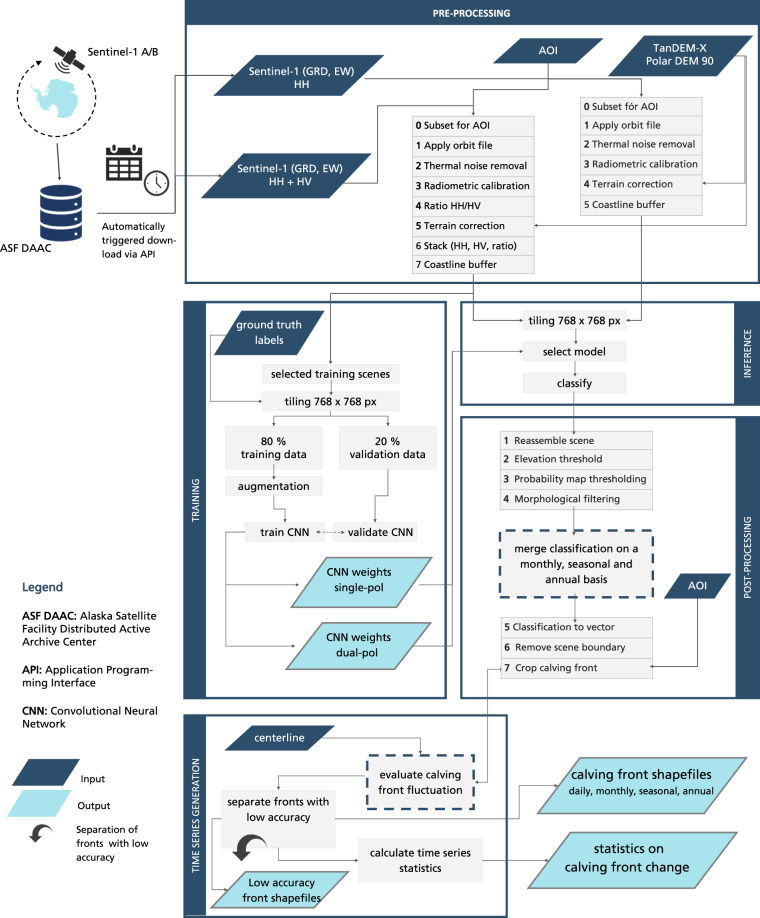
The IceLines workflow divided into satellite scene download, pre-processing, training of the convolutional neural network (CNN), inference for automated front extraction, post-processing of front predictions and the time series generation.

IceLines^24^ was developed to automatically monitor Antarctic ice shelf front positions and to deliver dense time series on calving front change. This requires a high degree of automatization as time-consuming manual front delineations cannot keep up with rapidly growing satellite archives^12^. Figure 2 shows the entire processing pipeline of IceLines which is subdivided into the following six steps: Data download, pre-processing of satellite data, training of the convolutional neural network (CNN), inference for automated front extraction, post-processing of front predictions and the time series generation. All individual processing steps are explained in the following.

### Download & pre-processing

On a monthly basis, available Sentinel-1 GRD EW scenes (40 m resolution) are downloaded for each monitored ice shelf defined by an area of interest (AOI) from the Alaska Satellite Facility Distributed Active Archive Center (ASF DAAC)^26^. We selected EW data as it provides more spatial context to the deep learning model at a defined tiles size (in pixels) and the data coverage for dual polarized EW scenes over the Antarctic coastline is better than for IW data. To avoid unnecessary high data amounts and the processing of scenes only partly covering selected fronts, the download is limited by filtering for suitable satellite orbits fully covering each front. Hence, the availability of daily front positions depends on Sentinel-1 acquisitions covering the front and can vary approximately from one to eight observations per month. Sentinel-1 data availability in Antarctica is limited before the adjustment of the acquisition scheme in May 2017 due to the launch of Sentinel-B and after its failure in December 2021. In cases where a front is never fully covered by an entire scene, adjacent scenes acquired on the same day are merged after pre-processing. The pre-processing is performed with the Graph Processing Tool of the ESA SNAP Toolbox^27^ on a Hadoop Cluster with 63 nodes (32/64 GB RAM) within a Docker container. For single polarized scenes, the pre-processing includes subsetting to the shelf extent, thermal noise removal, radiometric calibration, geometric terrain correction with the TanDEM-X PolarDEM 90 m^28^ and masking the imagery by a 100 km wide coastline buffer. Additionally, for dual polarized imagery, the ratio between the HH and HV polarization is calculated and added to the scene stack as described in Baumhoer *et al*. 2019 (ref. ^23^).

### Training & inference

The calving front is extracted with a HED-UNet which was initially developed by Heidler *et al*.^29^ and adapted for a circum-Antarctic utilisation in IceLines. The adaptation included a more diverse training data set with 81 Sentinel-1 scenes (instead of 11 scenes) covering 17 coastal regions (instead of only 2) and the ability of the network to automatically recognize and predict dual and single polarized imagery. This is necessary as Sentinel-1 imagery acquired before 2017 only includes one polarization. Moreover, for some coastal areas only single polarized imagery is available (e.g. Bellingshausen Sea) during the entire acquisition period of Sentinel-1.

The HED-UNet is a neural network architecture designed specifically for the extraction of calving fronts that builds on the two principles of segmentation and edge detection. While segmentation aims to differentiate glacier pixels from ocean pixels, edge detection directly classifies the pixels on the calving front. HED-UNet incorporates both approaches to encourage the model to learn visual patterns that are characteristic of the ice sheet, the ocean, and the calving zone. By working on multiple resolution levels, the model can consider a larger spatial context than previous models, which has been found important for the task of calving front detection. The training data covers the time period May 2017 until August 2019.

**Fig. 3:**
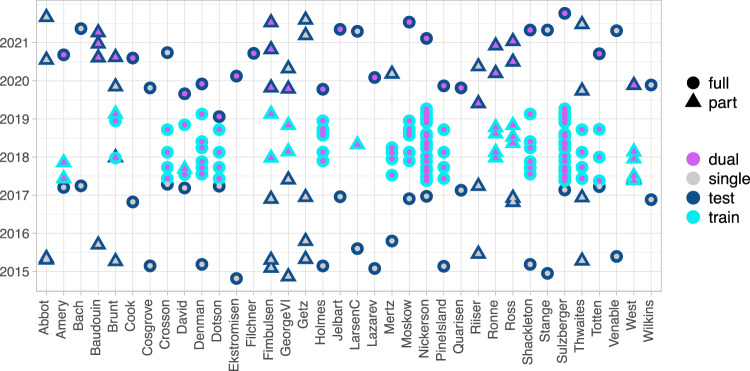
Distribution of training and validation data for each ice shelf or glacier. Training data is plotted in light blue, validation data in dark blue. Violet and grey dots indicate the polarization of the used satellite imagery. For training, the dual polarized image was additionally used to train weights for single polarized imagery by using only the first channel (HH polarization). Circles and triangles indicate whether the entire front or a part of it was used for training/validation.

Figure 3 shows the distribution of training data broken down for each individual ice shelf or glacier. Note that some ice shelves are covered by the same scene, hence, the training dates are the same for some ice shelves.

### Post-processing

The post-processing step uses the prediction probability of the HED-UNet segmentation merging head for the classes “ice sheet” and “ocean” where the border of both classes marks the coastline. An elevation threshold is applied on the prediction probability to exclude areas above the grounding line by assigning all areas above an empirically set elevation threshold of 110 m (TanDEM-X PolarDEM 90 m^28^ referenced to the WGS84 ellipsoid) to the class ice sheet. This threshold is also applicable for future front positions except entire ice shelves disintegrate in combination with drastic grounding line retreat. This removes erroneous classifications over the dry snow zone in high altitude ice sheet areas (sometimes mistaken with low backscatter areas over the ocean) but does not influence predictions over lower ice shelf areas. The elevation supported prediction probability is binarized by a threshold of 50% (66% for annual products derived from the seasonal products). Afterwards, morphological filtering is applied to ensure that both classes are contiguous areas. After obtaining the final classification result, temporal aggregation is applied by merging all available segmentation results per month, season and year. For monthly products the daily prediction probability is merged by averaging. For seasonal and annual products, the monthly and seasonal classifications are merged by averaging the binary masks, respectively.

IceLines provides calving front time series on different temporal scales as the temporal aggregation of predictions for several time steps allows more robust front delineations. For example, occasionally misplaced predictions over the ocean due to wind roughened sea, mélange, icebergs or sea ice are compensated by merging several classification results. The binarized classification result is used to extract a polygon from the area classified as ice sheet. The polygon shapefile is tested for validity (bounded area, no self-intersections, multipart geometries, try to get notches of polygon) before a line is extracted from the polygon. The line is clipped to remove the scene boundaries and retrieve the coastline. The clipping extent equals the extent of the initial satellite image eroded by 15 pixels. In case of monthly averaged products scene boundaries might still occur if scene extents varied within one month. Lastly, the coastline is clipped by the AOI for each monitored ice shelf or glacier to obtain the final calving front position.

### Time series generation

Deep neural networks demonstrated their superior performance for calving front detection in several studies in means of speed and still achieve accuracies better or comparable to manual delineations^21–23,29–31^. Nevertheless, occasional failures occur where incorrect front positions are extracted due to icebergs, mélange, surface melt or cloud cover. These erroneous fronts can easily be detected visually but the automatization of this process is still challenging. Either, unsuitable optical imagery (e.g. cloud cover, invisible front) can be eliminated in beforehand^21^ or the front itself can be verified by polygon complexity measures^22^. In our case, both approaches did not provide satisfying results which led us to the creation of a reliability measure based on the calving front time series. The calving front time series is calculated along perpendicular centerlines comparable to the centre-line method described in Lea *et al*.^32^. Instead of one single centerline we used several centerlines to better cover the ice shelf front and measure the sea-ward intersection if several intersections (e.g. due to crevasses) exist. The number (3 to 15 centerlines per shelf) and spacing (3 to 65 km) of centerlines varies depending on ice shelf size and complexity of the front. In rare cases, where ice shelf front areas are known to be repeatedly extracted ambiguously (e.g. at D-15 iceberg in front of West Ice Shelf, fast ice area in front of Filchner Ice Shelf) centerlines are either skipped or moved to obtain the actual movement pattern of the front not biased by these areas. Potentially incorrect front positions can be detected within the time series as they are either significantly land-inwards in case of surface melt or sea-wards due to mélange or sea ice cover. In order to detect theses outliers, it is presumed that a calving front position cannot deviate more than the mean plus/minus the standard deviation (minimum 2 pixels (80 meters) to account for certain degree of variation) of eight (four for monthly, seasonal and annual products) previous and successive front positions. The number of eight (four) front positions ensures to cover consecutive erroneous fronts e.g. during periods of surface melt. Most recent front positions at the end of the time series are considered again in the outlier detection during monthly processing to ensure that front positions of large calving events are finally not classified as outliers. In practice this means when searching for a front position of a recent calving event it is more likely to find it in the folder ‘fronts-eliminated’ within the first three months. Front positions classified as outliers are stored in a separate folder indicating the user to check these fronts visually. This approach works reliable for fronts with clear movement patterns of advance and retreat but often excludes too many confident front positions when frequent and smaller calving events happen especially for the daily product. The share between confident and potentially unreliable front detections varies between <1% and 99% depending strongly on ice shelf front geometry, periods of surface melt, fast ice areas and calving pattern. On average, 47.4% of the daily and 38.8% of the monthly front positions require further visual inspection.

## Data Records

The IceLines data set^24^ is available at DLR’s GeoService and updated on a monthly basis. Front positions are provided as line shapefiles for each ice shelf. In case of ice shelves being larger than the acquired Sentinel-1 scenes, the front positions are divided in sub-fronts and numbered as indicated in Fig. 1. Each folder includes shapefiles aggregated on a daily, monthly, seasonal and annual basis. They are separated into confidently extracted fronts (folder ‘fronts’) and fronts which require further manual checks before using them for further analysis (folder ‘fronts-eliminated’). The file naming convention for a daily, monthly, seasonal and annual product is:

Daily:[*polarization*]_[*YYYYMMDD*]_[*S1 unique product ID*]-[*ice shelf name*].gpkg

Monthly:[*polarization*]_[*YYYYMM*]-[*ice shelf name*].gpkg

Seasonal:[*YYYY*][*Quartal*]_mean-[*ice shelf name*].gpkg

Annual:[*YYYY*]noQ1_mean-[*ice shelf name*].gpkg

“1SDH” indicates that the front was extracted from a dual polarized input scene, “1SSH” from a single polarized one. The quarters are defined as austral seasons with Q1 summer (Dec, Jan, Feb), Q2 autumn (Mar, Apr, May), Q3 winter (Jun, Jul, Aug) and Q4 spring (Sep, Oct, Nov). The annual average excludes the summer season where mostly surface melt occurs (indicated by “noQ1” in the file name). The attribute table of each file includes the date of the front position (‘DATE_’), ice shelf name (‘name’), date of last update (‘updated’, yyyymmdd) and version number (‘version’). For daily and monthly products, the name of the corresponding Sentinel-1 scene is included (‘s1name’). The shapefiles are in Antarctic polar stereographic projection (EPSG:3031). All IceLines^24^ products can be downloaded from the DLR GeoService (https://download.geoservice.dlr.de/icelines/files/) as single GeoPackage or combined ZIP files for each temporal product and ice shelf separated in the folders ‘fronts’ and ‘fronts-eliminated. Moreover, visual exploration of annual and monthly calving front positions is possible via the web browser of the DLR GeoService (https://geoservice.dlr.de/web/maps/eoc:icelines).

## Technical Validation

IceLines is validated by calculating the distance difference between the automatically extracted daily fronts and manual front delineations. This is a commonly applied method to assess the accuracy of the derived front positions^21,23,31^. Nevertheless, manual front delineation is a very challenging task and underlies a high degree of subjectivity. Having several experts delineating the same front will always generate different results^12,30,33,34^. Hence, the automated front position is not necessarily wrong if it does not align with the manual reference. To perform a comprehensive validation including all kinds of different calving front morphologies and acquisition dates, 92 fronts were selected for an accuracy assessment. The selection criteria required two fronts for each ice shelf, one derived from single polarized imagery between October 2014 and April 2017 and one derived from dual polarized imagery between August 2018 and October 2021. If only single polarized imagery were available, two single polarized samples were taken. Both time periods were selected to lie outside the time span of the training data. The exact distribution of training and validation data is shown in Fig. 3. The validation dates were determined by taking a random date sample for each ice shelf and time period. From the confident front extractions, the front position available closest to the randomly selected date was taken for validation. It has to be mentioned that no confident front positions could be derived for Filchner, Ronne and parts of Ross and West ice shelves based on single polarized imagery. Hence, they could not be included for validation. That applies also for parts of Baudouin and Getz ice shelves as either no imagery was acquired during the first or second validation period, respectively. For the validation of bigger ice shelves, the front was subdivided into several parts (indicated by triangles in Fig. 3) to ensure that the extracted front was derived from only one clearly identifiable satellite scene. Taken together, all frontal parts cover the entire ice shelf front.

### Mean distance error

**Fig. 4:**
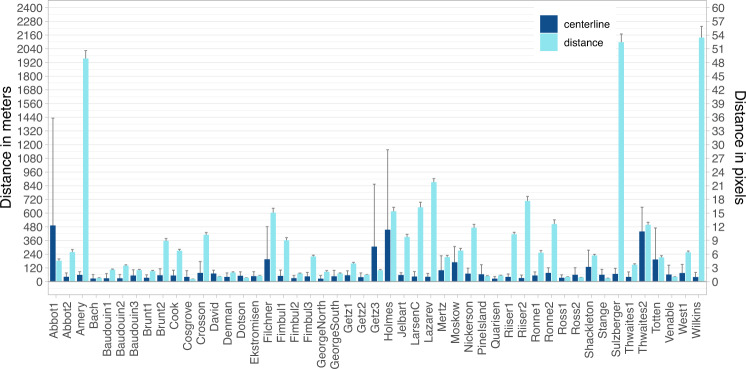
Validation accuracies for the IceLines data set. For each ice shelf and glacier, the mean distance and centerline accuracies with 95% confidence are shown.

**Fig. 5:**
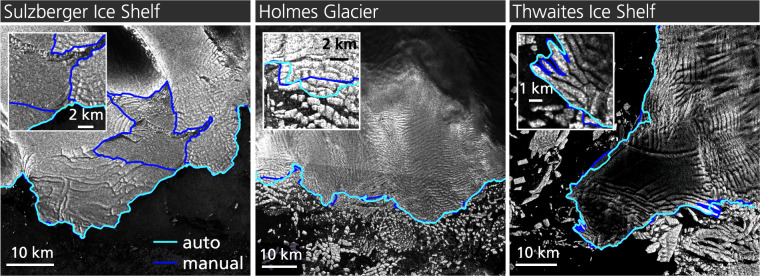
Automated (blue) and manually (violet) derived calving fronts for Sulzberger Ice Shelf, Holmes Glacier and Thwaites Ice Shelf. Copernicus Sentinel-1 Data 2022.

The distance difference between manual and automated fronts was tested by measuring the distance from the manual to the automated front along pixel spaced (40 m) points. This measure gives an impression how good the extracted front matches the manual delineation. Figure 4 shows the mean distance error for each ice shelf with 95% confidence in light blue. Additionally, all validation values are summarized in Table S1. The accuracy varies depending on the assessed calving front and input imagery polarization. For example, the mean distance accuracy for Amery and Sulzberger ice shelves is low as the front extraction with single polarized imagery was not able to detect fast ice areas as shown in Fig. 5. The front of Sulzberger Ice Shelf is extracted very accurately except for the vast (>30 km) fast ice area which increases the mean distance error significantly. Best mean distance accuracies were reached for Cosgrove, Bach and Stange ice shelves (<1 pixel). On average, the true mean distance error for fronts extracted from dual-pol imagery is 209 ± 12 m (5.2 pixel) with 95% confidence and 432 ± 21 m (8.8 pixel) for single polarized imagery which is comparable with existing studies on calving front extraction for Antarctica. Previous studies on CNN-based calving front detection published mean distance accuracies between 96.32 m (1.97 pixel)^34^, 86.76 ± 1.43 m (2.25 pixel)^21^, 38 m (~6 pixel)^30^ and 86 m^22^ for Greenland and 108 m (2.7 pixel)^23^, 222 ± 23 (Wilkes Land)^29^, 345 ± 24 (Antarctic Peninsula)^29^, 237.12 m^35^ and 330.63 m (2.35 pixel)^21^ for Antarctica. It should be noted that these accuracies are not directly comparable due to temporal and spatial variations in the validation data sets spanning from one^30,34^ to 62^21^ glaciers during different time periods. For further comparison, manual delineations from different experts can deviate between 92.5 m^34^, 33 m (5.5 pixel)^30^ and 183 m (4.6 pixel)^23^ depending on image resolution and difficulty of the calving front delineation.

### Frontal movement accuracy

To assess how accurately the frontal movement of a calving front can be determined with the IceLines data set^24^, the distance accuracy was calculated along perpendicular centerlines already utilized for the time series generation. The frontal movement accuracy is determined as the mean distance between the manual and automated front along the defined centerlines. Figure 4 shows the frontal movement accuracy in dark blue. Additionally, the results of the accuracy assessment are summarized in Table S2. On average, the error is 63 ± 68 m (1.6 pixel) for dual polarized imagery and 107 ± 126 m (2.7 pixel) for single polarized imagery with 95% confidence. Highest mean centerline inaccuracies exist for glaciers with frequent and small calving events (e.g. Holmes and Totten Glacier as well as Thwaites Glacier Tongue) due to difficulties to distinguish icebergs from crevassed glacier ice as shown for Holmes Glacier in Fig. 5. Besides certain challenging fronts, frontal movement can be assessed with high accuracy. For 84% of the calving fronts within the validation data set, the frontal movement can be assessed with an accuracy better than 2 pixels (<80 m) independent from the polarization of the input imagery.

### Validation summary

The comprehensive validation of IceLines demonstrates that CNN-derived calving fronts offer a good and accurate alternative to laborious manual front delineations. The front position can deviate from manual delineated fronts due to fast ice, mélange and icebergs close to the front by 209 ± 12 m (5.2 pixel) on dual polarized imagery and 432 ± 21 m (8.8 pixel) for single polarized imagery whereas the frontal movement can be determined with higher accuracies of 63 ± 68 m (1.6 pixel) for dual and 107 ± 126 m (2.7 pixel) for single polarized imagery. Limitations of the IceLines data set^24^ exist due to SAR specific backscatter properties. Calving front detections during the occurrence of surface melt or within the dry snow zone (e.g. Ronne, Ross and Filchner ice shelves) remain challenging especially with single polarized SAR imagery. But the benefits of illumination- and cloud-independent SAR imagery outweigh these limitations and do not significantly affect the unprecedented value of the IceLines data set^24^ represented by regularly updated, circum-Antarctic time series of calving front change.

**Fig. 6:**
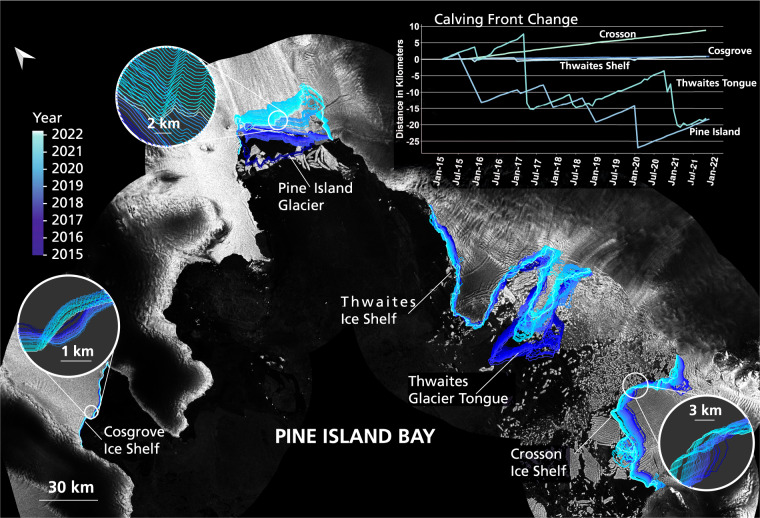
Calving front change in Pine Island Bay between 2015 and 2021 visualized with the monthly calving front positions of IceLines. The plot in the upper right shows the calving front time series for each shelf/glacier along a central centerline in flow direction. Copernicus Sentinel-1 Data 2022.

**Fig. 7:**
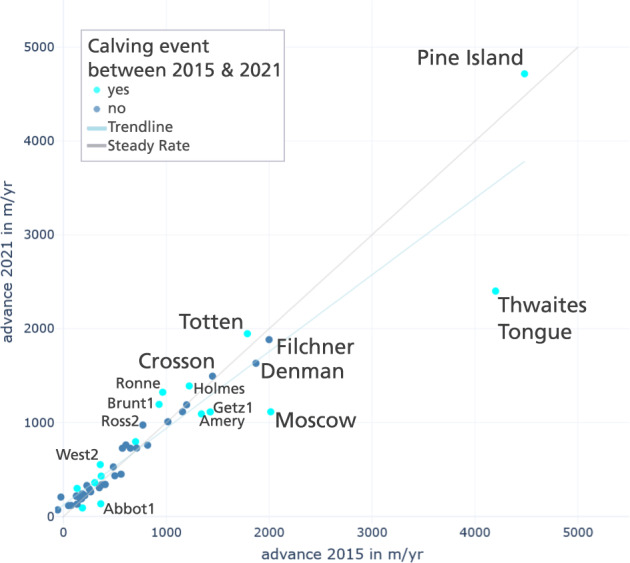
Advance rates of Antarctic ice shelves in 2015 compared to 2021. Based on all ice shelves, the blue trendline shows a slight tendency of higher advance rates for fast moving fronts in 2015. For reference, the grey line stands for a steady advance rate with no change between 2015 and 2021. Dots above the reference line show an accelerated advance rate in 2021 compared to 2015, dots below show the opposite. Dots in cyan indicate if the ice shelf calved in-between the advance in 2015 and 2021.

To conclude, we provide a final impression of the very detailed IceLines data set to show new possibilities for analysing calving front change with IceLines^24^. Figure 6 shows the monthly calving front change in Pine Island Bay between 2015 and 2021. The different calving front dynamics in Pine Island Bay become visible ranging from slight advance of the Cosgrove (+1.01 km) and Thwaites Ice Shelves (+0.78 km) over the steady advance of the Crosson Ice Shelf (+8.80 km), the strong retreat of Thwaites glacier tongue (−16.86 km) to the retreat and frequent calving of Pine Island Glacier (total change −19.05 km) between 2015 and 2021. Figure 7 shows the frontal advance rates of Antarctic ice shelves in 2015 and 2021 which were calculated along a perpendicular centerline on the basis of all reliable front positions within the respective year. If a calving event occurred within 2015 or 2021, the ice shelf front advance was calculated on the longer remaining advance time series. Pine Island Glacier has the highest advance rate with 4.72 km/yr in 2021 followed by Thwaites Tongue with 4.20 km/yr in 2015. Pine Island Glacier accelerated between 2015 and 2021 in contrast to the disintegrating Tongue of Thwaites. Based on all ice shelves, the linear regression trendline (R^2^ = 0.89) shows a slight tendency to higher advance rates in 2015 compared to 2021 for stronger advancing (>0.65 km /yr) shelves. It has to be mentioned that this trend is strongly dominated by the disintegrating glacier tongue of Thwaites not having a clear advancing front in 2021 compared to 2015. In contrast to this overall tendency, the frontal advance of Pine Island, Totten, Ross West, Ronne, Larsen C and Brunt1 ice shelves accelerated in 2021 compared to 2015. Furthermore, it is noteworthy that shelves with a calving event between 2015 and 2021 more likely changed their frontal advance rate compared to shelves without calving (see cyan dots in Fig. 7). For further exploration the supplementary includes Table S3 with all advance rates and an interactive and zoomable version of Fig. 7 can be accessed at https://download.geoservice.dlr.de/icelines/files/icelines_auxiliary_v1.zip.

## Usage Notes

The IceLines data set^24^ was created to provide scientists and modellers with up-to-date calving front positions without requiring time-consuming manual delineations. Even though for many fronts accuracies on pixel level can be achieved, some extracted fronts should be checked manually before use, especially those located in the folder “fronts-eliminated”. This can be done in a geographic information system (e.g. QGIS - qgis.org). Areas of failed front extraction can easily be identified by winding lines not located between previous and consecutive front positions. If in doubt, the front positions can be compared with the original Sentinel-1 imagery by the unique Sentinel-1 ID and acquisition date or the Sentinel-1 scene name in the attribute table of each file. Sentinel-1 imagery can be downloaded from ASF (search.asf.alaska.edu) and ESA (scihub.copernicus.eu). Pre-processed Sentinel-1 imagery can be accessed via Google Earth Engine (earthengine.google.com). Moreover, the probability of a failed front extraction decreases with higher temporal aggregation. This means that temporal aggregated products (e.g. monthly, seasonal, annual) should be preferred over daily products. The IceLines data set is registered under the DOI 10.15489/btc4qu75gr92.

## Supplementary information

Supplementary

## Data Availability

The processing of IceLines has been carried out in the Calvalus system and GPU-cluster available at DLR’s Earth Observation Center by means of proprietary software and dedicated Python (v3.6 and v3.7) scripts. Given the use of proprietary tools, the implemented processing pipeline cannot be openly released to the public. However, all processing steps can be accessed and reproduced as follows: The pre-processing of the Sentinel-1 imagery can be replicated with the open source ESA SNAP Toolbox 8.0 and the processing steps described in Fig. 2. The code of the HED-Unet based on Pytorch (v1.7) is available at https://github.com/khdlr/HED-UNet and the final post-processing script (Python v3.7) is available at https://download.geoservice.dlr.de/icelines/files/icelines_auxiliary_v1.zip. Additionally, this folder includes data used for the accuracy assessment, a Python script for bulk download (bulk-download-icelines.py) and an exemplary script (‘display-icelines-gee.js’) to display IceLines data with the corresponding Sentinel-1 scenes in Google Earth Engine.

## References

[1] Rignot E, Jacobs S, Mouginot J, Scheuchl B (2013). Ice-Shelf Melting Around Antarctica. Science.

[2] Fürst JJ (2016). The safety band of Antarctic ice shelves. Nat. Clim. Change.

[3] Cook AJ (2016). Ocean forcing of glacier retreat in the western Antarctic Peninsula. Science.

[4] Cook AJ, Fox AJ, Vaughan DG, Ferrigno JG (2005). Retreating glacier fronts on the Antarctic Peninsula over the past half-century. Science.

[5] Cook AJ, Vaughan DG (2010). Overview of areal changes of the ice shelves on the Antarctic Peninsula over the past 50 years. The Cryosphere.

[6] Greene, C. A., Gardner, A. S., Schlegel, N.-J. & Fraser, A. D. Antarctic calving loss rivals ice-shelf thinning. *Nature* (2022). 10.1038/s41586-022-05037-w 35948639

[7] Baumhoer CA, Dietz AJ, Kneisel C, Paeth H, Kuenzer C (2021). Environmental drivers of circum-Antarctic glacier and ice shelf front retreat over the last two decades. The Cryosphere.

[8] Davies BJ, Carrivick JL, Glasser NF, Hambrey MJ, Smellie JL (2012). Variable glacier response to atmospheric warming, northern Antarctic Peninsula, 1988–2009. The Cryosphere.

[9] Doake CSM, Vaughan DG (1991). Rapid disintegration of the Wordie Ice Shelf in response to atmospheric warming. Nature.

[10] Jenkins A (2018). West Antarctic Ice Sheet retreat in the Amundsen Sea driven by decadal oceanic variability. Nat. Geosci..

[11] Miles BWJ, Stokes CR, Jamieson SSR (2016). Pan–ice-sheet glacier terminus change in East Antarctica reveals sensitivity of Wilkes Land to sea-ice changes. Sci. Adv..

[12] Baumhoer CA, Dietz AJ, Dech S, Kuenzer C (2018). Remote Sensing of Antarctic Glacier and Ice-Shelf Front Dynamics—A Review. Remote Sens..

[13] Lovell AM, Stokes CR, Jamieson SSR (2017). Sub-decadal variations in outlet glacier terminus positions in Victoria Land, Oates Land and George V Land, East Antarctica (1972–2013). Antarct. Sci..

[14] Marshall SJ (2005). Recent advances in understanding ice sheet dynamics. Earth Planet. Sci. Lett..

[15] Wuite J (2019). Sub-Annual Calving Front Migration, Area Change and Calving Rates from Swath Mode CryoSat-2 Altimetry, on Filchner-Ronne Ice Shelf, Antarctica. Remote Sens..

[16] Frezzotti M (1997). Ice front fluctuation, iceberg calving flux and mass balance of Victoria Land glaciers. Antarct. Sci..

[17] Liu Y (2015). Ocean-driven thinning enhances iceberg calving and retreat of Antarctic ice shelves. Proc. Natl. Acad. Sci..

[18] Sohn H-G, Jezek KC (1999). Mapping ice sheet margins from ERS-1 SAR and SPOT imagery. Int. J. Remote Sens..

[19] Liu H, Jezek KC (2004). Automated extraction of coastline from satellite imagery by integrating Canny edge detection and locally adaptive thresholding methods. Int. J. Remote Sens..

[20] Wu SY, Liu AK (2003). Towards an automated ocean feature detection, extraction and classification scheme for SAR imagery. Int. J. Remote Sens..

[21] Cheng D (2021). Calving Front Machine (CALFIN): glacial termini dataset and automated deep learning extraction method for Greenland, 1972–2019. The Cryosphere.

[22] Zhang E, Liu L, Huang L, Ng KS (2021). An automated, generalized, deep-learning-based method for delineating the calving fronts of Greenland glaciers from multi-sensor remote sensing imagery. Remote Sens. Environ..

[23] Baumhoer CA, Dietz AJ, Kneisel C, Kuenzer C (2019). Automated Extraction of Antarctic Glacier and Ice Shelf Fronts from Sentinel-1 Imagery Using Deep Learning. Remote Sens..

[24] Baumhoer C (2022). EOC GeoService.

[25] Secretariat SCAR (Scientific Committee on Antarctic Research). *Composite Gazetteer of Antarctica. GCMD Metadata 1992 (updated 2014 and 2017)* https://data.aad.gov.au/aadc/gaz/scar/ (2017).

[26] Alaska Satellite Facility. *Distributed Active Archive Center (ASF DAAC).* https://search.asf.alaska.edu/#/ (2022).

[27] ESA SNAP - Sentinel Application Platform, version 8.0. http://step.esa.int (2020).

[28] Wessel B (2021). TanDEM-X PolarDEM 90 of Antarctica: generation and error characterization. The Cryosphere.

[29] Heidler, K., Mou, L., Baumhoer, C., Dietz, A. & Zhu, X. X. HED-UNet: Combined Segmentation and Edge Detection for Monitoring the Antarctic Coastline. *IEEE Trans. Geosci. Remote Sens*. 1–14 (2021).

[30] Zhang E, Liu L, Huang L (2019). Automatically delineating the calving front of Jakobshavn Isbræ from multitemporal TerraSAR-X images: a deep learning approach. The Cryosphere.

[31] Loebel, E. *et al*. Extracting glacier calving fronts by deep learning: the benefit of multi-spectral, topographic and textural input features. *IEEE Trans. Geosci. Remote Sens*. 1–1 (2022).

[32] Lea JM, Mair DWF, Rea BR (2014). Evaluation of existing and new methods of tracking glacier terminus change. J. Glaciol..

[33] Goliber S (2022). TermPicks: a century of Greenland glacier terminus data for use in scientific and machine learning applications. The Cryosphere.

[34] Mohajerani Y, Wood M, Velicogna I, Rignot E (2019). Detection of Glacier Calving Margins with Convolutional Neural Networks: A Case Study. Remote Sens..

[35] Holzmann, M. *et al*. Glacier Calving Front Segmentation Using Attention U-Net. in *2021 IEEE International Geoscience and Remote Sensing Symposium IGARSS* 3483–3486 (IEEE, 2021).

