# Supplementary material for: IceLines – A new data set of Antarctic ice shelf front positions
Source: Sci Data. 2023 Mar 15;10:138. doi: 10.1038/s41597-023-02045-x (PMC10017676; doi:10.1038/s41597-023-02045-x)
Supplement: Supplementary file 1 — Supplementary [file 41597_2023_2045_MOESM1_ESM.pdf]

Table S1: Mean distance error between manual and automated fronts.

	Date	Shelf	mean	median	max	min	std	No. measured points	margin	polarization
1	27/12/2017	Brunt1	108.63	21.52	3003.22	0.01	358.59	4657	10.30	dual
2	24/01/2019	Dotson	33.75	28.80	138.03	0.04	25.60	1214	1.44	dual
3	29/05/2019	Riiser2	618.17	37.26	7050.40	0.03	1540.14	6580	37.21	dual
4	31/08/2019	David	48.53	39.82	384.55	0.00	41.66	4827	1.18	dual
5	13/10/2019	Holmes	249.44	82.01	2083.81	0.04	359.61	2988	12.89	dual
6	16/10/2019	GeorgeNorth	31.57	24.44	161.59	0.03	27.01	746	1.94	dual
7	26/10/2019	Quarisen	31.95	21.78	429.05	0.01	39.33	2541	1.53	dual
8	27/10/2019	Fimbul1	262.97	42.50	3256.10	0.01	591.51	5287	15.94	dual
9	15/11/2019	PineIsland	22.60	16.58	239.40	0.02	22.29	1872	1.01	dual
10	20/11/2019	West1	422.34	50.59	6467.69	0.00	982.48	10263	19.01	dual
11	03/12/2019	Denman	47.92	35.61	310.03	0.21	48.57	1111	2.86	dual
12	03/02/2020	Lazarev	561.79	67.36	5839.03	0.06	1165.60	8258	25.14	dual
13	16/02/2020	Ekstromisen	66.13	33.60	1133.97	0.03	116.26	7751	2.59	dual
14	07/03/2020	Mertz	296.49	33.41	4227.15	0.00	744.16	4046	22.93	dual
15	13/03/2020	Ronne1	252.36	42.22	5598.65	0.01	879.43	7300	20.17	dual
16	30/06/2020	Ross1	34.21	21.07	957.64	0.01	65.26	4051	2.01	dual
17	07/08/2020	Cook	288.75	69.84	2755.97	0.01	519.61	5341	13.94	dual
18	10/08/2020	Baudouin2	139.13	28.74	2140.16	0.00	333.32	7121	7.74	dual
19	13/08/2020	Brunt1	72.97	36.76	1710.26	0.00	166.11	4855	4.67	dual
20	07/09/2020	Amery	606.44	77.39	11438.62	0.01	1659.44	13745	27.74	dual
21	17/09/2020	Totten	146.93	38.46	1870.09	0.04	317.65	2464	12.54	dual
22	21/09/2020	Filchner	603.22	43.45	9200.20	0.01	1576.73	5848	40.41	dual
23	27/10/2020	Fimbul3	96.31	36.82	2304.55	0.02	242.77	7593	5.46	dual
24	02/12/2020	Ronne2	504.27	62.59	11823.12	0.03	1754.20	8607	37.06	dual
25	20/12/2020	Baudouin1	104.45	36.45	1545.79	0.00	215.06	5767	5.55	dual
26	13/01/2021	Ross2	34.57	26.22	182.73	0.00	31.41	6009	0.79	dual
27	11/02/2021	Nickerson	85.81	49.51	1292.31	0.00	114.09	8056	2.49	dual
28	07/04/2021	Baudouin3	41.85	23.25	874.34	0.01	69.08	8024	1.51	dual
29	02/05/2021	Shackleton	150.65	38.30	3736.92	0.01	434.22	10804	8.19	dual
30	09/05/2021	Jelbart	409.45	42.81	4589.99	0.02	900.59	4814	25.44	dual
31	12/07/2021	Fimbul2	58.17	25.90	1301.42	0.00	132.88	5281	3.58	dual
32	10/08/2021	Moskow	192.96	59.94	2035.69	0.01	351.42	2691	13.28	dual
33	09/10/2021	Sulzberger	280.49	61.64	6090.93	0.00	759.16	7994	16.64	dual
34	27/10/2014	Ekstromisen	35.20	21.53	682.05	0.00	54.11	6791	1.29	single
35	13/11/2014	GeorgeSouth	83.42	26.58	2957.01	0.00	300.73	4746	8.56	single
36	15/12/2014	Stange	22.44	16.61	169.55	0.03	21.10	2631	0.81	single
37	31/01/2015	Lazarev	1179.71	143.81	7794.66	0.00	1853.30	8277	39.93	single
38	03/02/2015	Fimbul1	459.77	53.71	7187.91	0.03	1232.67	5773	31.80	single
39	21/02/2015	PineIsland	71.77	21.19	1674.23	0.00	207.86	2094	8.90	single
40	24/02/2015	Holmes	981.70	126.97	7107.15	0.01	1793.72	3811	56.95	single
41	25/02/2015	Cosgrove	22.44	15.72	374.51	0.04	27.04	1360	1.44	single
42	11/03/2015	Denman	111.47	51.33	694.85	0.01	139.32	884	9.18	single
43	11/03/2015	Shackleton	305.83	26.39	6172.80	0.01	783.12	10680	14.85	single
44	08/04/2015	Brunt2	365.61	51.83	6126.97	0.01	952.09	9188	19.47	single
45	13/04/2015	Thwaites2	231.98	62.97	2467.34	0.00	442.28	5179	12.05	single

46	21/04/2015	Fimbul2	83.79	35.78	1134.14	0.00	138.68	5508	3.66	single
47	22/04/2015	Abbot2	294.59	27.65	5294.58	0.00	866.79	4762	24.62	single
48	29/04/2015	Getz1	275.14	59.95	4441.75	0.02	658.58	5163	17.96	single
49	08/05/2015	Abbot1	320.97	25.55	4187.85	0.02	853.32	2960	30.74	single
50	26/05/2015	Venable	35.03	27.12	207.15	0.01	29.14	2500	1.14	single
51	18/06/2015	Riiser1	478.20	55.88	5189.84	0.00	963.08	9600	19.27	single
52	09/08/2015	LarsenC	75.66	20.21	3749.29	0.00	335.81	8678	7.07	single
53	14/09/2015	Baudouin3	158.11	32.77	4264.23	0.00	533.85	6915	12.58	single
54	18/10/2015	Getz3	162.29	24.15	1456.58	0.03	316.22	2448	12.53	single
55	21/10/2015	Mertz	131.29	51.09	1555.83	0.00	239.79	3879	7.55	single
56	25/10/2016	Ross2	38.84	29.79	361.64	0.00	34.44	7069	0.80	single
57	29/10/2016	Cook	251.83	49.12	2235.85	0.04	473.36	5416	12.61	single
58	20/11/2016	Wilkins	597.09	45.62	5629.76	0.00	1210.18	5947	30.76	single
59	25/11/2016	Fimbul3	343.13	45.41	6080.01	0.01	894.40	8767	18.72	single
60	29/11/2016	Moskow	349.65	74.39	3623.38	0.02	681.15	2750	25.46	single
61	05/12/2016	Ross1	47.29	31.57	758.62	0.02	75.55	3883	2.38	single
62	09/12/2016	Thwaites1	138.80	45.13	1272.08	0.00	235.73	3316	8.02	single
63	12/12/2016	Getz2	59.18	41.90	950.92	0.04	73.54	3440	2.46	single
64	18/12/2016	Jelbart	371.34	63.68	3338.17	0.03	729.51	4779	20.68	single
65	22/12/2016	Nickerson	857.47	68.33	17622.76	0.00	2743.11	8688	57.68	single
66	18/02/2017	Quarisen	73.77	24.80	983.26	0.00	141.45	2483	5.56	single
67	21/02/2017	Sulzberger	3914.93	97.84	22214.99	0.00	5762.24	7928	126.84	single
68	12/03/2017	David	39.19	27.34	460.72	0.00	45.10	4745	1.28	single
69	17/03/2017	Amery	3303.13	79.47	30613.22	0.00	7126.25	15956	110.57	single
70	25/03/2017	Totten	276.08	70.86	2510.78	0.04	488.64	2490	19.19	single
71	28/03/2017	Riiser2	796.09	40.81	7635.98	0.01	1624.59	6238	40.32	single
72	29/03/2017	Dotson	32.10	26.25	223.42	0.08	25.71	1256	1.42	single
73	01/04/2017	Bach	40.77	22.70	862.51	0.03	97.90	1108	5.76	single
74	16/04/2017	Crosson	279.79	119.22	2443.08	0.07	423.30	3318	14.40	single
75	29/05/2017	GeorgeNorth	140.06	31.51	1718.42	0.02	302.99	953	19.24	single
76	03/06/2017	West1	90.32	34.56	1253.04	0.00	160.33	9453	3.23	single
77	27/09/2019	Thwaites1	155.65	41.66	1716.56	0.03	300.86	3841	9.51	single
78	26/10/2019	Cosgrove	20.94	16.28	139.44	0.02	18.04	1356	0.96	single
79	06/11/2019	Brunt2	347.04	30.37	8847.61	0.01	1162.38	10159	22.60	single
80	23/11/2019	Wilkins	3678.70	55.00	20955.21	0.02	6367.13	5539	167.68	single
81	17/05/2020	Riiser1	352.25	50.96	4777.84	0.00	732.38	10174	14.23	single
82	19/07/2020	Abbot2	222.44	26.56	4434.13	0.01	724.16	4983	20.11	single
83	28/09/2020	Crosson	538.79	161.13	4106.22	0.04	856.98	3622	27.91	single
84	12/10/2020	GeorgeSouth	56.69	25.90	1330.47	0.00	134.51	6022	3.40	single
85	11/03/2021	Getz1	42.30	24.53	647.24	0.00	61.54	4222	1.86	single
86	20/04/2021	LarsenC	1225.08	57.42	23501.18	0.02	3954.01	8633	83.41	single
87	26/04/2021	Venable	47.82	21.74	781.03	0.01	107.32	2536	4.18	single
88	02/05/2021	Stange	36.25	23.60	580.78	0.01	53.03	2774	1.97	single
89	16/05/2021	Bach	21.82	15.90	116.87	0.00	21.07	1164	1.21	single
90	24/06/2021	Thwaites2	762.78	325.40	5186.26	0.02	1009.48	3637	32.81	single
91	07/08/2021	Getz3	35.89	28.74	385.06	0.01	31.61	2585	1.22	single
92	30/08/2021	Abbot1	44.00	23.56	743.78	0.02	77.21	2884	2.82	single

Table S2: Frontal Movement Accuracy Assessment

	Date	Shelf	mean	mean_abs	max	min	std	std_abs	margin	polarization
1	27/12/2017	Brunt1	3.41	31.55	50.17	-78.47	39.72	21.70	25.95	dual
2	24/01/2019	Dotson	-23.51	45.52	46.92	-126.08	60.84	43.33	48.68	dual
3	29/05/2019	Riiser2	11.48	31.02	67.66	-39.03	37.20	20.38	27.56	dual
4	31/08/2019	David	96.62	96.62	134.08	30.17	43.83	43.83	38.42	dual
5	13/10/2019	Holmes	332.00	396.45	2073.01	-89.51	855.22	821.63	684.32	dual
6	16/10/2019	GeorgeNorth	2.32	29.18	40.25	-36.17	34.93	12.73	30.62	dual
7	26/10/2019	Quarisen	-33.49	33.49	-12.20	-73.38	24.60	24.60	21.56	dual
8	27/10/2019	Fimbul1	-14.85	46.69	79.89	-167.10	72.22	54.56	50.05	dual
9	15/11/2019	PineIsland	-30.26	69.43	44.51	-173.96	100.62	70.12	98.61	dual
10	20/11/2019	West1	-7.22	52.61	182.23	-119.87	82.31	61.30	51.02	dual
11	03/12/2019	Denman	11.44	40.55	50.76	-67.05	47.68	21.40	38.15	dual
12	03/02/2020	Lazarev	4.94	45.70	103.87	-70.40	56.68	29.22	39.27	dual
13	16/02/2020	Ekstromisen	-3.36	70.31	149.23	-103.82	91.73	53.53	59.93	dual
14	07/03/2020	Mertz	-29.87	62.31	64.88	-97.89	68.25	25.91	66.89	dual
15	13/03/2020	Ronne1	-53.25	53.25	-12.66	-91.38	32.21	32.21	31.57	dual
16	30/06/2020	Ross1	-20.15	25.56	6.76	-59.46	31.59	25.86	30.96	dual
17	07/08/2020	Cook	59.84	59.84	94.27	32.42	28.47	28.47	27.90	dual
18	10/08/2020	Baudouin2	28.36	28.62	93.33	-0.66	37.77	37.52	33.10	dual
19	13/08/2020	Brunt1	21.68	34.95	78.01	-28.23	39.78	27.14	25.99	dual
20	07/09/2020	Amery	65.74	65.74	130.68	35.37	40.15	40.15	32.13	dual
21	17/09/2020	Totten	-41.28	47.99	20.11	-95.36	40.90	30.90	32.73	dual
22	21/09/2020	Filchner	-138.09	195.64	175.36	-988.79	386.45	356.28	286.29	dual
23	27/10/2020	Fimbul3	12.80	42.52	95.28	-64.94	53.08	30.00	39.32	dual
24	02/12/2020	Ronne2	-76.21	76.21	-36.46	-130.14	45.75	45.75	44.83	dual
25	20/12/2020	Baudouin1	-3.79	28.31	36.78	-37.41	37.58	15.22	42.53	dual
26	13/01/2021	Ross2	-27.95	50.73	45.56	-75.22	52.71	19.70	51.65	dual
27	11/02/2021	Nickerson	-44.03	79.44	113.01	-422.87	122.14	101.17	61.81	dual
28	07/04/2021	Baudouin3	18.47	43.79	115.82	-54.93	55.83	35.75	41.36	dual
29	02/05/2021	Shackleton	17.09	37.71	74.82	-56.27	44.22	24.49	35.39	dual
30	09/05/2021	Jelbart	-3.58	24.94	37.58	-50.45	32.35	18.34	23.97	dual
31	12/07/2021	Fimbul2	-14.72	23.41	34.77	-46.94	24.66	15.14	17.09	dual
32	10/08/2021	Moskow	-19.41	53.95	69.09	-83.18	63.79	26.27	62.51	dual
33	09/10/2021	Sulzberger	24.70	52.69	150.23	-55.55	67.34	45.39	46.67	dual
34	27/10/2014	Ekstromisen	-10.00	24.72	47.51	-41.79	28.13	14.70	18.38	single
35	13/11/2014	GeorgeSouth	-1.08	45.10	50.62	-112.61	65.30	41.52	57.24	single
36	15/12/2014	Stange	-2.39	44.69	78.12	-69.12	55.49	27.50	41.11	single
37	31/01/2015	Lazarev	-36.25	37.25	3.99	-85.91	30.18	28.76	20.92	single
38	03/02/2015	Fimbul1	-32.97	53.46	45.52	-180.34	74.05	58.82	51.32	single
39	21/02/2015	PineIsland	-31.73	58.89	28.55	-117.45	71.45	42.70	70.02	single
40	24/02/2015	Holmes	-386.08	512.85	201.63	-2161.10	896.21	816.36	717.12	single
41	25/02/2015	Cosgrove	-23.03	47.89	35.13	-125.42	71.47	52.52	70.04	single
42	11/03/2015	Denman	-33.37	40.67	21.91	-93.74	40.71	31.75	32.57	single
43	11/03/2015	Shackleton	191.58	220.41	765.80	-51.56	322.80	299.91	258.29	single
44	08/04/2015	Brunt2	-17.93	41.59	32.96	-81.45	51.83	31.56	41.47	single
45	13/04/2015	Thwaites2	-65.94	65.94	-23.27	-125.64	50.49	50.49	49.48	single
46	21/04/2015	Fimbul2	-16.82	35.49	44.44	-90.24	42.07	25.56	29.15	single

47	22/04/2015	Abbot2	-10.57	50.78	112.63	-151.70	71.75	49.35	42.40	single
48	29/04/2015	Getz1	-61.87	62.74	3.06	-146.40	50.36	49.08	37.31	single
49	08/05/2015	Abbot1	-930.05	932.70	3.66	-4628.35	2067.46	2065.96	1812.21	single
50	26/05/2015	Venable	-8.12	16.52	31.53	-38.93	20.30	13.26	14.07	single
51	18/06/2015	Riiser1	-34.49	43.44	32.73	-126.94	45.88	36.57	27.11	single
52	09/08/2015	LarsenC	-3.03	26.92	47.80	-45.50	34.76	18.70	27.81	single
53	14/09/2015	Baudouin3	15.49	62.58	127.32	-124.25	80.35	46.55	59.52	single
54	18/10/2015	Getz3	570.35	570.35	1102.60	38.10	752.71	752.71	1043.21	single
55	21/10/2015	Mertz	-6.82	135.93	141.54	-285.49	190.86	108.88	187.05	single
56	25/10/2016	Ross2	34.29	67.55	146.99	-82.87	84.27	53.56	73.87	single
57	29/10/2016	Cook	-6.74	47.06	32.79	-107.60	67.40	40.63	66.06	single
58	20/11/2016	Wilkins	-8.57	37.48	75.99	-50.31	45.54	22.84	33.74	single
59	25/11/2016	Fimbul3	-45.41	48.35	11.77	-125.02	43.37	39.57	30.06	single
60	29/11/2016	Moskow	-284.00	284.00	-118.32	-585.16	218.83	218.83	214.45	single
61	05/12/2016	Ross1	-41.59	41.59	-17.96	-64.66	21.03	21.03	20.61	single
62	09/12/2016	Thwaites1	-25.98	30.06	7.55	-54.34	29.90	24.67	26.21	single
63	12/12/2016	Getz2	-11.72	38.33	58.92	-78.58	47.09	24.90	37.68	single
64	18/12/2016	Jelbart	-88.97	88.97	-55.07	-122.94	24.67	24.67	18.27	single
65	22/12/2016	Nickerson	-27.41	58.80	112.39	-159.81	69.01	43.16	34.92	single
66	18/02/2017	Quarisen	-8.16	17.13	17.56	-28.27	19.25	9.33	16.87	single
67	21/02/2017	Sulzberger	47.85	80.83	119.37	-69.61	75.00	27.83	51.97	single
68	12/03/2017	David	44.33	44.33	74.33	15.99	22.73	22.73	19.92	single
69	17/03/2017	Amery	51.88	51.88	85.66	12.17	30.36	30.36	24.29	single
70	25/03/2017	Totten	-157.68	338.67	239.23	-1472.64	650.13	561.13	520.22	single
71	28/03/2017	Riiser2	20.20	30.12	80.58	-23.02	36.12	26.88	26.76	single
72	29/03/2017	Dotson	57.05	57.05	93.91	24.64	25.59	25.59	20.47	single
73	01/04/2017	Bach	-1.01	20.09	38.16	-20.14	26.87	13.60	26.33	single
74	16/04/2017	Crosson	18.80	38.44	77.39	-34.45	48.86	29.81	47.88	single
75	29/05/2017	GeorgeNorth	-6.25	22.04	26.20	-38.18	28.04	15.09	24.58	single
76	03/06/2017	West1	-62.15	98.27	135.91	-400.90	161.03	139.62	99.81	single
77	27/09/2019	Thwaites1	9.84	50.71	129.94	-52.94	72.27	46.14	63.35	single
78	26/10/2019	Cosgrove	-11.56	34.16	45.21	-43.34	39.88	14.58	39.09	single
79	06/11/2019	Brunt2	8.14	69.55	93.19	-137.10	88.41	45.72	70.74	single
80	23/11/2019	Wilkins	-8.34	41.32	51.19	-143.68	64.84	47.90	48.04	single
81	17/05/2020	Riiser1	-18.59	38.11	53.28	-82.33	45.42	29.08	26.84	single
82	19/07/2020	Abbot2	-13.58	33.82	37.90	-95.42	42.79	27.85	25.29	single
83	28/09/2020	Crosson	11.33	114.33	222.37	-127.52	155.13	82.50	152.02	single
84	12/10/2020	GeorgeSouth	26.02	48.29	82.92	-55.68	53.73	28.59	47.09	single
85	11/03/2021	Getz1	-40.84	48.14	24.70	-121.49	56.55	49.40	41.89	single
86	20/04/2021	LarsenC	50.18	60.60	153.27	-26.05	68.77	57.33	60.28	single
87	26/04/2021	Venable	53.14	107.69	583.37	-61.10	217.14	192.67	150.47	single
88	02/05/2021	Stange	71.89	73.65	197.87	-3.30	76.91	74.94	56.97	single
89	16/05/2021	Bach	0.98	32.68	42.27	-60.78	45.14	24.78	44.23	single
90	24/06/2021	Thwaites2	-812.65	812.65	-239.92	-1038.61	382.78	382.78	375.12	single
91	07/08/2021	Getz3	-43.36	43.36	-17.38	-69.33	36.74	36.74	50.91	single
92	30/08/2021	Abbot1	-2.51	50.98	60.56	-132.27	75.89	50.18	66.52	single

Table S3: Advance rates of ice shelf fronts 2015 and 2021 as shown in Figure 7.

shelf	rate2015|mean	rate2021|mean	rate2015|std	rate2021|std
Abbot1	362.97	135.70	479.52	939.32
Abbot2	207.45	224.28	577.34	403.83
Amery	1341.20	1092.89	434.29	312.76
Bach	-55.07	71.12	125.71	305.10
Baudouin1	303.93	360.72	261.42	206.80
Baudouin2	176.66	190.44	284.76	364.89
Baudouin3	132.50	130.74	397.46	286.91
Brunt1	930.19	1195.12	241.81	198.15
Brunt2	1157.28	1115.65	357.12	381.79
Cook	818.29	759.62	450.96	712.29
Cosgrove	193.34	233.78	427.44	339.73
Crosson	1448.04	1495.06	824.34	267.23
David	711.52	728.33	355.77	326.75
Denman	1871.41	1631.74	874.44	564.70
Dotson	367.04	430.38	339.15	348.69
Ekstromisen	227.32	329.76	471.36	182.08
Filchner	1999.30	1883.28	933.03	452.22
Fimbul1	133.65	300.32	424.69	637.55
Fimbul2	76.88	117.76	393.26	181.24
Fimbul3	650.26	727.56	463.99	253.87
GeorgeNorth	182.96	242.73	399.17	203.89
GeorgeSouth	348.90	307.14	469.38	281.06
Getz1	1426.39	1114.22	836.27	362.77
Getz2	500.11	433.82	478.68	406.03
Getz3	560.26	450.64	339.64	176.33
Holmes	1222.76	1390.99	1045.88	1055.70
Jelbart	573.74	727.07	101.62	349.90
LarsenC	701.19	797.25	686.14	654.53
Lazarev	265.13	263.91	253.07	224.68
Mertz	1197.29	1190.54	610.63	469.01
Moskow	2015.60	1115.35	641.18	928.17
Nickerson	49.98	116.20	389.94	400.57
PineIsland	4480.77	4716.42	1226.11	554.99
Quarisen	166.96	186.51	329.76	137.04
Riiser1	371.50	338.07	1042.80	313.27
Riiser2	127.36	215.15	442.97	163.99
Ronne1	964.92	1323.41	652.67	634.49
Ross1	485.92	528.61	209.82	352.03
Ross2	772.21	974.56	158.00	735.00
Ross3	1015.26	1009.29	383.69	951.79
Shackleton	406.81	341.63	409.11	302.98
Stange	127.88	220.40	428.14	371.27
Sulzberger	-23.96	207.64	313.91	269.43
Thwaites1	182.86	237.87	397.85	370.64
Thwaites2	4200.05	2400.80	1499.66	832.59
Totten	1787.79	1946.83	383.14	419.61

Venable	250.74	294.28	401.62	194.66
West1	606.65	762.17	613.24	320.16
West2	358.06	553.26	562.45	308.13
Wilkins	184.61	91.62	178.73	360.37

For Quarisen, Cosgorve, Ross East (Ross3) and George South ice shelves also data form the end of 2014 and beginning of 2016 was used due to little data available in 2015. For Ronne and Filchner ice shelves the reference year is 2017/2018 (instead of 2015) as no reliable data is available from single polarized scenes earlier. If a calving event occurred within 2015 or 2021, the ice shelf front advance was calculated on the longer remaining advance time series (e.g. calving in March, advance measured from April to December).
Advance calculated between the first and last reliable front position in m/yr.
Standard deviation (m/yr) based on rates calculated on a monthly basis meaning that a standard deviation within 480 m in one year account for frontal advance rate variations within one pixel (40 m) per month (12 x 40 m = 480 m).
